# Carotid artery dissection linked to intermittent apnoeic swimming: A case–control study

**DOI:** 10.1113/EP093047

**Published:** 2025-07-26

**Authors:** Damian M. Bailey, Richard G. Davies, Brenig L. Gwilym, Benjamin S. Stacey, Danny Walmsley, Michael H. Lewis, Josip Butkovic, Ivan Mumlek, Brad Perry, Zvonomir Vrselja, Shigehiko Ogoh, James A. Pawelczyk, Mohamad Bashir, Richard D. White, Ian M. Williams

**Affiliations:** ^1^ Neurovascular Research Laboratory, Faculty of Life Sciences and Education University of South Wales Glamorgan UK; ^2^ Bexorg, Inc. New Haven Connecticut USA; ^3^ Department of Anaesthetics University Hospital of Wales Cardiff UK; ^4^ Department of Vascular Surgery University Hospital of Wales Cardiff UK; ^5^ Department of Biomedical Engineering Toyo University Asaka Japan; ^6^ Noll Physiological Research Center, Department of Kinesiology Penn State University University Park Pennsylvania USA; ^7^ Vascular and Endovascular Surgery, Health & Education Improvement Wales Cardiff UK; ^8^ Department of Radiology University Hospital of Wales Cardiff UK

**Keywords:** apnoea, carotid artery dissection, exercise, stroke

## Abstract

Internal carotid artery (ICA) dissection is a rare and potentially devastating cause of cerebral ischaemia, initiated by an intimal tear or rupture of the vasa vasorum, that can lead to an intraluminal thrombus, vascular stenosis, occlusion, or dissecting aneurysm formation. Management is challenging due to its complex pathophysiology and non‐specific nature of symptoms. In this case–control study, we were able to document the clinical presentation and management of an ICA dissection in a hypertensive, 50‐year‐old male triathlete following an acute bout of intermittent apnoeic (pyramid breathing) swimming. He developed blurred vision in his left eye, ipsilateral headache, pulsatile tinnitus and later noticed left‐sided ptosis and pupil miosis consistent with Horner's syndrome, prompting specialist referral. Neuroimaging confirmed a dissection of the left ICA and incidental pseudoaneurysm of the distal right ICA. The patient recovered well due to a combination of pharmacological/dietary management of hypertension and graduated, structured return to physical activity and competition, culminating in significant re‐expansion of the ICA true lumen calibre. We also conducted a laboratory‐based, dry‐land, static swimming simulation in an age‐ and physical activity‐matched healthy male control. This demonstrated that exercise‐induced ICA shear stress was more exaggerated during dynamic apnoeic breathing compared to normal breathing, which, in the setting of the patient's hypertension, may have been a precipitating factor underlying ICA dissection. Collectively, these findings provide unique insights into the pathophysiology and management of this rare condition while highlighting the inherent risks associated with this mode of exercise training in susceptible individuals with hypertension.

## Abstract (highlights)

- **What is the main observation in this case?**
  This case–control study describes the clinical presentation and treatment of a hypertensive competitive male triathlete who suffered an internal carotid artery (ICA) dissection after intermittent apnoeic (pyramid breathing) swimming. Dry‐land, static swimming simulated in a healthy male control demonstrated that this mode of exercise caused an exaggerated elevation in ICA shear stress, which may have been a precipitating factor.
- **What insights does it reveal?**
  These findings highlight the inherent risks in this mode of exercise and provide unique insights into the pathophysiology and management of this rare and potentially devastating cause of cerebral ischaemia.

## 1 INTRODUCTION

Dissections of the cervical (carotid and vertebral) arteries are uncommon, albeit potentially devastating conditions, that contribute to 2% of all ischaemic strokes (Bejot et al., 2014) and up to 25% of ischaemic stroke in adults under 50 years of age (Ekker et al., 2023). They associate with local signs and symptoms including headache and neck pain, cranial neuropathies, Horner's syndrome, pulsatile tinnitus and pseudoaneurysm formation (Blum & Yaghi, 2015). The annual incidence rate is approximately three cases per 100,000 although this is likely an underestimation due to asymptomatic cases (Lee et al., 2006). More than 50% of cases occur spontaneously, with up to 90% of dissections occurring in response to minor trauma caused by chiropractic neck manipulations, heavy lifting, whiplash, childbirth, vomiting, coughing, sneezing and vigorous exercise (Engelter et al., 2013).

The pathogenesis of cervical artery dissection is multifactorial, involving the complex interplay between risk factors, environmental triggers, genetic or congenital factors including connective tissue disorders, and anatomical factors including an elongated styloid process or increased vascular tortuosity (Yaghi, Engelter et al., 2024). It is typically caused by an intimal tear or rupture of the vasa vasorum, that can lead to an intraluminal thrombus, vascular stenosis, occlusion, or dissecting aneurysm formation. The initial insult is blood entering the vessel wall through a tear in the intima/media layer (Figure 1).

**FIGURE 1:**
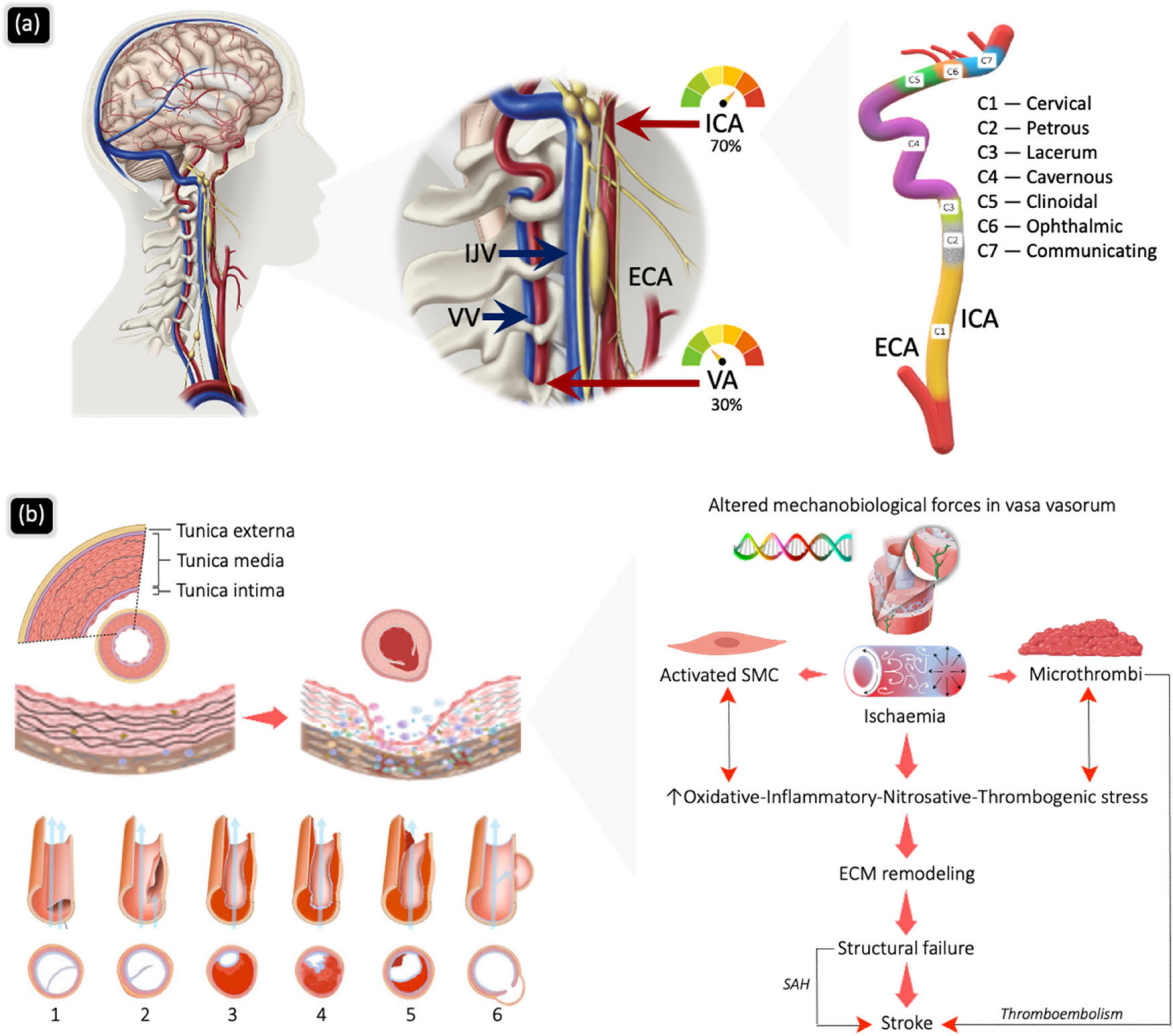
Arterial supply to the brain and pathophysiology of cervical arterial dissection. (a) Arterial supply to the brain highlighting anatomical distribution of the internal (ICA) and external (ECA) carotid and vertebral (VA) arteries relative to the internal jugular (IJV) and vertebral (VV) veins. Cervical and intracranial segments of the ICA are illustrated according to Bouthillier's classification (Bouthillier et al., 1996). (b) Pathophysiological mechanisms underlying cervical arterial dissection involving complex interaction between environmental and genetic risk determinants. The arterial dissection is typically initiated by a sudden (spontaneous or traumatic) tear in the intimal layer with subsequent bleeding into the subintimal space. Propagation of the tear creates a false (double) lumen leading to stenosis or occlusion, often with thrombus formation at the site of dissection. Dissection into the subadventitial layers causes (pseudo)aneurysmal dilatation of the artery wall. Images 1–6 highlight typical anatomical evolution of arterial dissections illustrating the appearance of: (1) double lumen; (2) intimal flap; (3) intramural haematoma; (4) irregular surface; (5) intraluminal thrombus; (6) pseudoaneurysm. Figure adapted from Wu et al. (2019). Constitutional risk factors combined with complex changes in blood flow topology involving viscous and inertial forces and/or microthrombi‐mediated cerebral ischaemia‐induced activation of the oxidative–inflammatory–nitrosative–thrombogenic stress pathways adversely impact arterial wall integrity. These can result in impaired smooth muscle cell metabolism, disruption of the extracellular matrix (ECM) and cytoskeletal dysfunction (Bailey et al., 2023). Stroke can occur either as a direct consequence of cerebral ischaemia due to true lumen narrowing/occlusion or release of microemboli. SAH, sub‐arachnoid haemorrhage (intracranial dissection). Figure created with BioRender.com.

In this case–control study, we had the unique opportunity to follow in detail the case of an exercise‐induced internal carotid artery (ICA) dissection in a hypertensive competitive male triathlete following an acute bout of intermittent apnoeic (pyramid breathing) swimming. We document the preceding events, clinical presentation, pathophysiology and management of this rare and potentially devastating cause of cerebral ischaemia, while highlighting the inherent risks associated with this mode of exercise training in susceptible individuals.

## 2 METHODS

### 2.1 Design

This study adopted a case‐control design. The case involved a male patient who experienced an ICA dissection potentially linked to swimming exercise that incorporated dynamic apnoeic breathing, a form of intermittent ‘hypoxic’ training (see section 2.3, Study 1 – Case). The control involved a healthy, age‐ and physical activity‐matched male participant who performed an acute bout of dry‐land, static apnoeic swimming designed to replicate the patient's training activity to determine its cerebrovascular consequences.

### 2.2 Ethical approval

Both patient and participant provided verbal and written informed consent. For the healthy participant who volunteered for the simulation sub‐study, all investigative procedures were approved by the Research Ethics Committees of the University of South Wales (no. 241411HR) and conformed to the most recent (7th) amendment of the *Declaration of Helsinki* of the World Medical Association (WMA, 2013) – with the exception that it was not registered in a publicly accessible database.

### 2.3 Study 1 – Case

The patient was a 50‐year‐old male amateur triathlete and consultant anaesthetist, with a body mass of 78 kg, 1.73 m tall and body mass index (BMI) of 26.1 kg/m^2^. He had a magnetic resonance (MR) angiogram of the renal arteries requested by cardiology 11 years ago due to a history of hypertension, which was normal. He also had a computed tomography (CT) coronary angiogram 9 years ago for a false positive electrocardiogram during a cardiopulmonary exercise test, which was also normal. His hypertension and migraines were treated with an angiotensin II receptor blocker, candesartan (16 mg once daily). His previous year of training data from global positioning data obtained via Strava included 3400 km of running, cycling and swimming with a cumulative elevation of 37,500 m. Cardiopulmonary exercise testing (CPET) on a cycle ergometer to volitional exhaustion conducted according to established procedures (Rose et al., 2018) 9 months prior, demonstrated a peak oxygen uptake (${\dot{V}}_{O_{2}\mathrm{peak}}$) of 53.3 mL/kg/min and hypertensive response to exercise (Figure 2a). In preparation for an upcoming triathlon event, he undertook dynamic intermittent apnoeic (pyramid breathing) swim training in a 50 m pool consisting of 500 m sets of front crawl swimming with breathing constrained to every 1/3/5/7/9/7/5/3/1 strokes involving bilateral neck rotations. This contrasted with his standard practice of 500 m sets of front crawl swimming breathing every two strokes, favouring his right side (contralateral to the ICA dissection).

**FIGURE 2:**
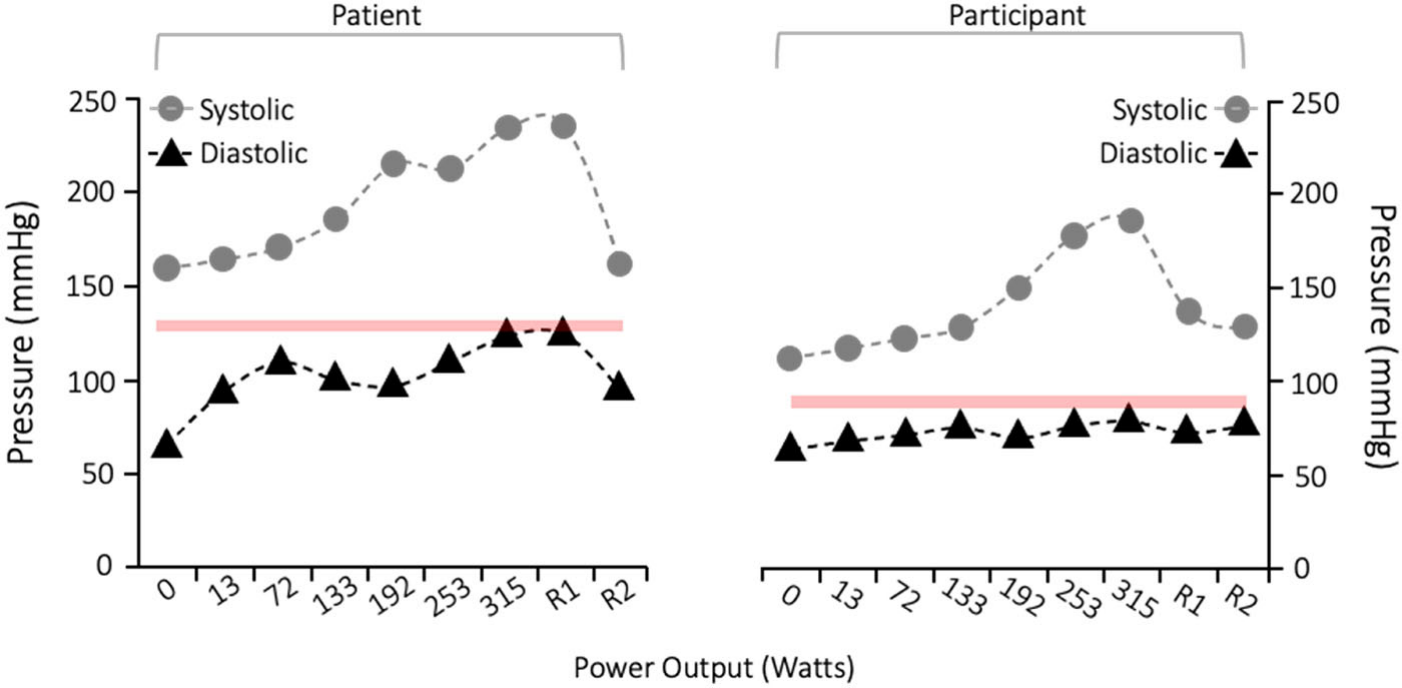
Blood pressure responses to exercise. Comparative visual highlighting the hypertensive response to a standardised cardiopulmonary exercise test (CPET) to volitional exhaustion in the patient. R1, first recovery period (immediate post exercise to 1 min 35 s); R2, second recovery period (1 min 35 s to 3 min 10 s). Red lines refer to the average mean arterial pressure recorded over the course of each CPET.

#### 2.3.1 Timeline of ICA dissection

Immediately following the pyramid swim training session, the patient developed blurred vision in the left eye that was accompanied by a diffuse left‐sided headache. Twenty minutes later, he developed a sudden popping sensation in his left ear followed by onset of pulsatile tinnitus. Two days later, a clinical colleague diagnosed left‐sided ptosis and pupil miosis consistent with Horner's syndrome (Figure 3). He immediately refrained from all physical activity (see section 2.3.3., Follow‐up and outcome).

**FIGURE 3:**
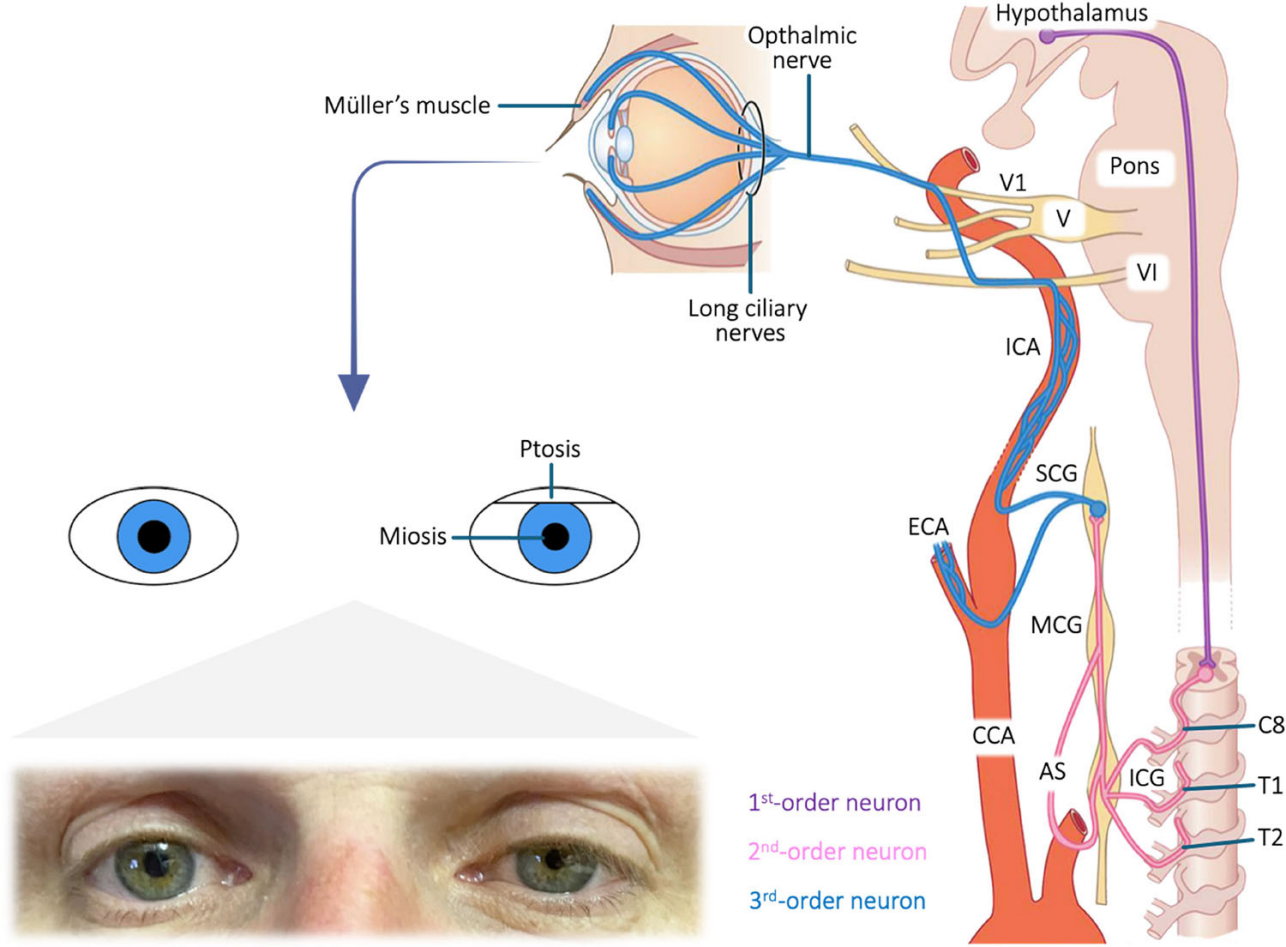
Horner's syndrome and the oculosympathetic pathway. AS, ansa subclavia; CCA, common carotid artery; ECA, external carotid artery; ICA, internal carotid artery; ICG, inferior cervical ganglion; MCG, middle cervical ganglion; SCG, superior cervical ganglion. Note patient's classic clinical triad of (left‐sided) symptoms characterised by eyelid ptosis (drooping), miosis (pupillary constriction), and facial anhidrosis (decreased sweating). Figure adapted from Reede et al. (2008).

#### 2.3.2 Neuroimaging

An urgent CT of the head, with CT angiography of the head and neck vessels, was performed within 2 days of initial symptoms. This demonstrated severe narrowing of the left ICA lumen immediately below the skull base and in the carotid canal in the petrous temporal bone, with appearances in keeping with dissection (Figure 4a). An incidental pseudoaneurysm of the distal right ICA was also identified, likely reflecting a previous (undetected) dissection (Figure 4a). The left ICA lumen was smaller than the right ICA in the carotid canal and cavernous sinus, with smaller calibre A1 and proximal M1 segments of the left anterior and middle cerebral arteries noted compared with the right side (Figure 4b). There was no evidence of cerebral infarction.

**FIGURE 4:**
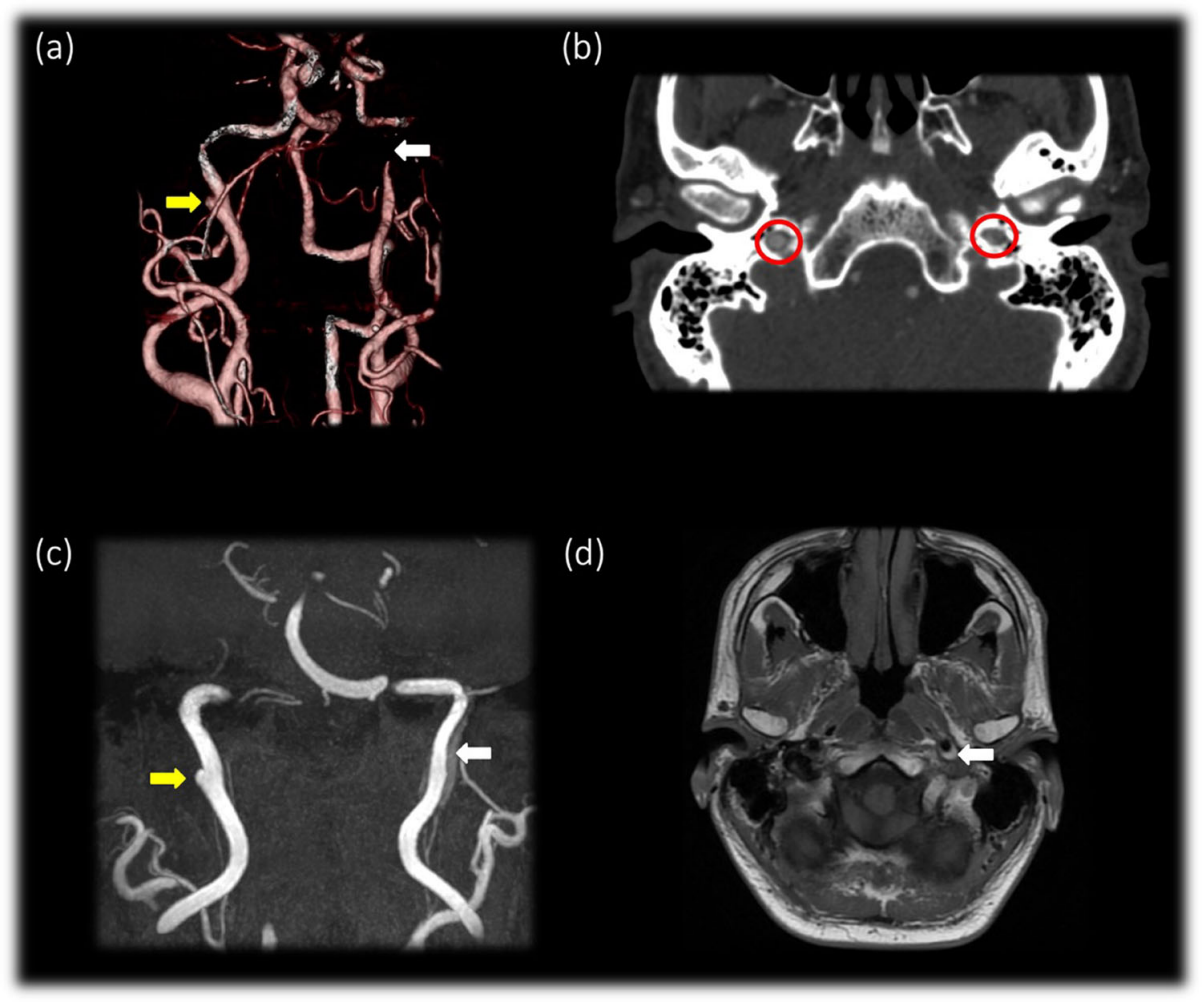
Neuroimaging findings highlighting internal carotid artery dissection. (a) 3D maximum‐intensity projection (MIP) computed tomography angiography (CTA) coronal oblique – illustrating left internal carotid artery (ICA) dissection (white arrow) and right ICA pseudoaneurysm (yellow arrow). (b) Axial CTA showing asymmetry of the ICAs in the carotid canal (red circles). (c) Coronal MIP magnetic resonance angiography time of flight image confirming left ICA dissection (white arrow) and right ICA pseudoaneurysm (yellow arrow). (d) Axial magnetic resonance imaging scan (in phase T1 Flex) confirmed the presence of a non‐flow‐limiting dissection of the left ICA, displaying the classical ‘crescent sign’ of dissection, secondary to intimal haemorrhage (white arrow). Note (a, b) recorded within 2 days of initial symptoms and (c, d) ∼6 weeks post.

Magnetic resonance imaging of the brain with angiographic sequences (MRA) was performed approximately 6 weeks post‐injury and confirmed the presence of a non‐flow‐limiting dissection of the left ICA (Figure 4c), with T1 fat saturation images highlighting the classical ‘crescent sign’ of dissection, secondary to intimal haemorrhage (Figure 4d). Flow was observed in the petrous cavernous and supraclinoid ICAs, with circle of Willis seen to be unremarkable. No intracranial aneurysm or vascular abnormality was identified.

#### 2.3.3 Follow‐up and outcome

Urgent referral to a neurologist prompted initiation of anti‐platelet treatment consisting of 300 mg of aspirin and 75 mg of clopidogrel for 2 weeks then clopidogrel alone for 12 months. A cardiologist optimised his hypertension control by increasing his candesartan dose to 24 mg once daily and commencing the β‐blocker, nebivolol (2.5 mg once daily).  His average arterial blood pressure on 24‐h recording with these medications was 125/85 mmHg. Lipid–lipoprotein profile indicated raised triglyceride (3.2 mmol/L) and low high‐density lipoprotein cholesterol (HDLc, 1.0 mmol/L) levels.

Two weeks later he developed severe, recurring left sided retro‐orbital pain at night, consistent with cluster headaches. To further control his hypertension, he commenced a strict low carbohydrate diet avoiding ultra‐processed foods aiming to consume <130 g/day of carbohydrates. Two weeks after commencing this regime, his arterial blood pressure was further reduced to 100/62 mmHg on 24‐h monitoring and body mass decreased to 71.2 kg, with a BMI of 23.8 kg/m^2^. During this 3‐month period post‐dissection, exercise was limited to gentle walking (only) for a few kilometres each day. He then gradually returned to low intensity aerobic exercise on a static cycle ergometer or outdoor jogging for a further 3 months aiming to keep heart rate (HR) at 100–120 b/min (Engelter, Tranenka, Grond‐Ginsbach et al., 2021). He continues to swim but has been advised not to engage in pyramid breathing or weight training, activities typically accompanied by intermittent Valsalva manoeuvres (see section 4.2, Pathophysiology).

A follow‐up MRA at 3 months demonstrated improvement of the ICA stenosis to approximately 75% of (normal) luminal calibre (i.e., 25% stenosis). Significant, albeit incomplete recovery of the extracranial circulation was also confirmed via bilateral Duplex ultrasound (Terason uSmart 3300, Teratech, Burlington, MA, USA) according to established procedures (Bailey et al., 2024) of the common carotid (CCA), ICA and vertebral (VA) arteries (Figure 5). During the subsequent 6 months, cycling and running distances and intensities were gradually increased without any neurological complications. The patient returned to the pool during this period, initially training only with a front‐mounted swimming snorkel to avoid neck rotation and after a further 6 months swam without it.

**FIGURE 5:**
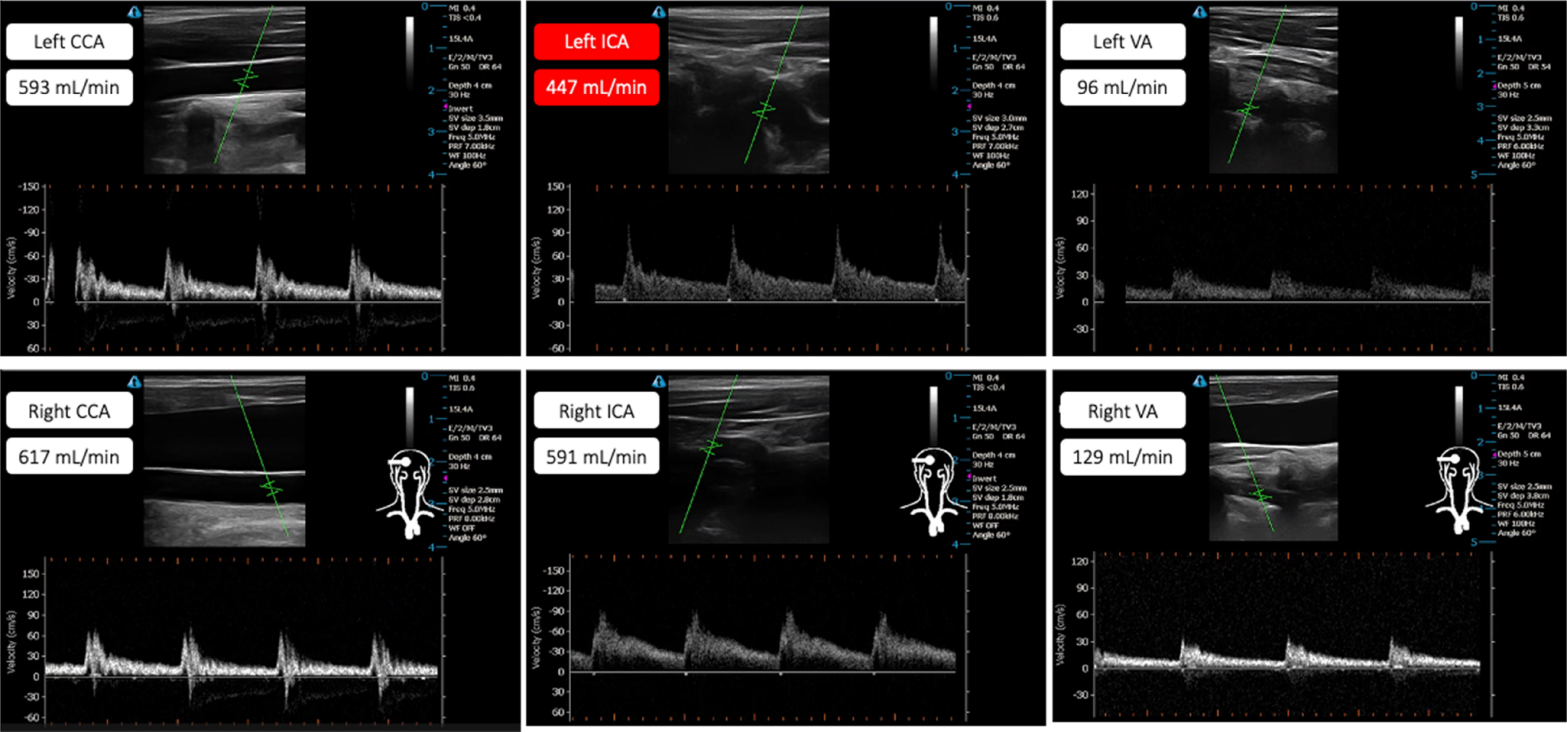
Duplex ultrasound images highlighting partial recovery of anterior and posterior extracranial blood flow in the patient. CCA, common carotid artery; ICA, internal carotid artery; VA, vertebral artery. Red shading highlights (incomplete) recovery perfusion in the (left) dissected ICA.

The patient continued with the low carbohydrate diet and was able to discontinue nebivolol and reduce the candesartan dose to 16 mg OD. A year later, blood pressure control was much improved with a mean of 114/73 mmHg. Fasting lipid profile demonstrated normal triglyceride (1.5 mmol/L) and HDLc (1.4 mmol/L) levels. He had a residual miosis but no other sequelae. Two years later, at a major UK triathlon Olympic distance event, he finished 21st overall and second in his age group and continues to engage with high‐volume endurance‐based training incorporating swimming, cycling and running.

### 2.4 Study 2 – Case control

A 54‐year‐old male amateur cyclist and University academic was recruited with a body mass of 64 kg, 1.67 m tall and BMI of 22.9 kg/m^2^. A recent medical examination confirmed that he was free of cardiovascular, pulmonary and cerebrovascular disease and was not taking any nutritional supplements including over‐the‐counter antioxidant or anti‐inflammatory medications. Blood sampling indicated a normal lipid–lipoprotein profile (total cholesterol: 3.92 mmol/L; triglycerides: 1.04 mmol/L; HDLc: 1.4 mmol/L; and LDLc: 2.0 mmol/L).

His previous year of training data from Strava registered 9997 km of cycling with a total elevation of 122,324 m. He also engaged in weight training 3–4 times/week. Cardiopulmonary exercise testing on a cycle ergometer using the identical ramped protocol undertaken by the patient demonstrated a ${\dot{V}}_{O_{2}\mathrm{peak}}$ of 64.9 mL/kg/min and normotensive response to exercise (Figure 2b).

#### 2.4.1 Design

Resting measurements were performed after 5 min in the prone position in the overnight fasted state with the head supported in the horizontal position (offsetting lack of buoyancy). Following familiarisation with the experimental set‐up, the participant was randomly assigned to perform two 5‐min bouts of simulated (i.e. dry‐land static) swimming with breathing constrained to every 1/3/5/7/9/7/5/3/1 strokes (pyramid breathing) and every 2 strokes (standard breathing) with each bout completed at the same stroke rate (1/sec) and interspersed by 30 min of passive recovery. Cardiopulmonary measurements were recorded continuously whereas ICA flow ($\dot{Q}$_ICA_) was determined during the final minute of each bout (Figure 6).

**FIGURE 6:**
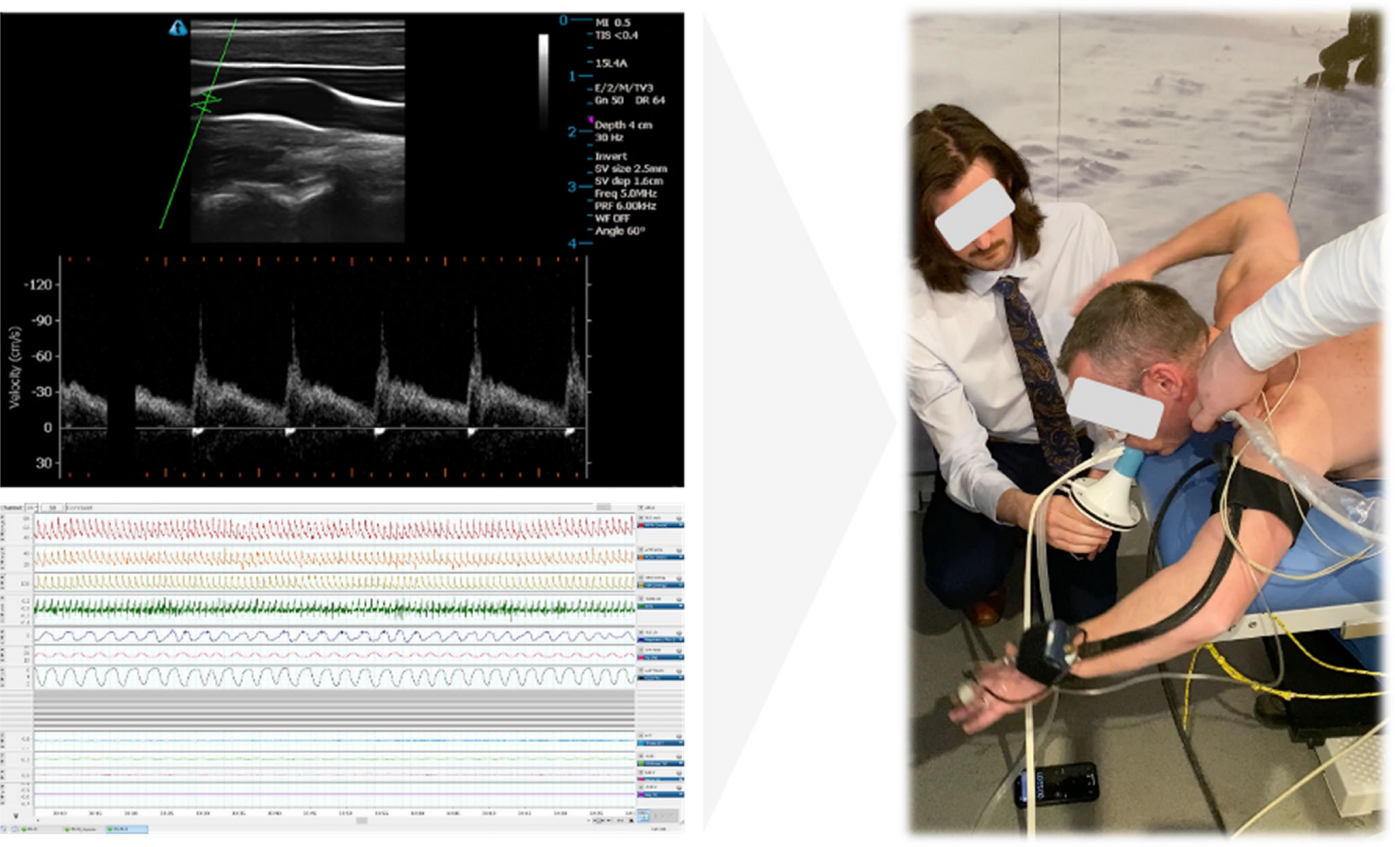
Laboratory‐based, dry‐land, static swimming experimental set‐up. Visual highlighting integrative physiological assessment of cardiopulmonary and cerebrovascular responses to simulated swimming incorporating both pyramid breathing (constrained to every 1/3/5/7/9/7/5/3/1 strokes) and standard breathing (constrained to every two strokes). Note typical data outputs in LabChart and Duplex images of the internal carotid artery.

#### 2.4.2 Haematology

Blood was obtained without stasis from an indwelling cannula located in a forearm antecubital vein into Vacutainers (Becton, Dickinson and Company, Oxford, UK) before immediate centrifugation at 600 *g* (4°C) for 10 min. Plasma was decanted into cryogenic vials (Nalgene Labware, Thermo Fisher Scientific Inc., Waltham, MA, USA) and stored at −80°C. Samples were defrosted and plasma glucose assayed photometrically (Randox Daytona Plus, Randox, Crumlin, UK). A separate sample of whole blood was assayed photometrically for haemoglobin (Hb, HemoCue 201+, Radiometer Limited, UK). Samples were assayed in triplicate and the average calculated. The intra‐ and inter‐assay coefficients of variation (CV) were <5%. Whole blood viscosity (η) at a shear rate of 225/s was measured in vitro at 37°C using a cone and plate viscometer (DV2T Viscometer, Brookfield Amtek, Chandler, AZ, USA) according to established methods (Gnasso et al., 2001).

#### 2.4.3 Cardiopulmonary function

HR was assessed using a three‐lead electrocardiogram (ECG, ADI BioAmp ML132, ADInstruments, Colorado Springs, CO, USA). Beat‐by‐beat arterial blood pressure was assessed via finger photoplethysmography and arterial volume clamping (Finometer PRO, Finapres Medical Systems, Amsterdam, Netherlands) and used to calculate mean arterial pressure (MAP) after calibrating values to the average of three automated brachial blood pressure measurements (Life Source, A&D Medical, Ann Arbor, MI, USA, model: UA767FAM), taken over a 5 min resting baseline period. Stroke volume (SV) was estimated from the arterial blood pressure waveform using the Modelflow (MF) algorithm (Wesseling et al., 1993), which incorporates participant/patient sex, age, stature and mass (BeatScope 1.0 software; TNO; TPD Biomedical Instrumentation, Amsterdam, The Netherlands). Peripheral oxygen saturation ($S_{pO_{2}}$) was measured using fingertip pulse oximetry (Nonin 9550 Onyx II, Nonin Medical, Inc., Plymouth, MI, USA). Respiratory flow was measured with a pneumotachometer (model HR 800 L, Hans Rudolph, Shawnee, KS, USA) and end‐tidal partial pressures of $CO_{2}$ ($P_{\mathrm{ETC}O_{2}}$) was sampled continuously via capnography (model ML206, ADInstruments).

#### 2.4.4 Cerebrovascular function

Separate assessments of diameter, velocity and blood flow recordings in the left and right common carotid, ICA and VA ($\dot{Q}$_CCA_ $/\dot{Q}$_ICA_/$\dot{Q}$_VA_) were obtained using a 10 MHz, multifrequency, linear array vascular ultrasound (Terason uSmart 3300) transducer according to established procedures (Thomas et al., 2015). Arterial diameter was measured via B‐mode imaging, whereas peak blood velocity was simultaneously measured with pulse‐wave mode. The CCA and ICA were imaged at least 1.5 cm below and above the carotid bifurcation, respectively, with no evidence of turbulent or retrograde flow. The VA was insonated at the C4–C5 or C5–C6 vertebral segment. The steering angle was fixed to 60° and the sample volume was placed in the centre of the vessel and adjusted to cover the entire vascular lumen. All images were recorded as video files at 30 Hz and stored for offline analysis using Cardiovascular Suite (Quipu, Pisa, Italy). Simultaneous measures of arterial diameter and velocity over 1 min were used to calculate flow.

#### 2.4.5 Data integration and analysis

All variables were sampled continuously at 1 kHz using an analog‐to‐digital converter (Powerlab, 16/30; ADInstruments) and data were interfaced with LabChart (Version 7.3), and analysed offline.

#### 2.4.6 Calculations

##### Cardiopulmonary

Cardiac output ($\dot{Q}$) was calculated as HR×SV.

Total peripheral resistance (TPR) was calculated as $\frac{\mathrm{MAP}}{\dot{Q}}$ where $\mathrm{MAP}\;=\;\mathrm{Diastolic}\;\mathrm{blood}\;\mathrm{pressure}\;+\frac{1}{3}\left(\mathrm{Systolic}\;\mathrm{blood}\;\mathrm{pressure}\;-\;\mathrm{Diastolic}\;\mathrm{blood}\;\mathrm{pressure}\right)$.

Rate pressure product [RPP; ×10^2^ in arbitrary units (AU)] was calculated as systolic BP × HR.

##### Cerebrovascular

Volumetric blood ($\dot{Q}$_CCA_ $\dot{Q}$_ICA_/$\dot{Q}$_VA_, mL/min) was calculated offline as:

$$\frac{{\text{CCA/ICA/VA-V}}_{P}\left(\mathrm{cm}/s\right)}{2}\times \pi {\left(\frac{\text{CCA/ICA/VA-Diameter (cm)}}{2}\right)}^{2}\times 60$$

where *V*_p_ is peak envelope blood velocity.

Cerebrovascular conductance index (ICA‐CVCi, mL/min/mmHg) was calculated as:

$$\frac{{\dot{Q}}_{\mathrm{ICA}\;(\mathrm{mL}/\min)}}{\mathrm{MAP}\;(\mathrm{mmHg})}$$

Estimated arterial oxygen content ($C_{aO_{2}}$, mL/dL) was estimated as:

$$\left(\mathrm{Hb}\;(g/\mathrm{dL})\;\times \;1.34\;\times \;\frac{{S_{pO_{2}}}_{\;(\%)\;}}{100}\right)+[0.003\;\times P_{aO_{2}}\;(\mathrm{mmHg})]$$

assuming arterial partial pressure of O_2_ ($P_{aO_{2}}$) = 100 mmHg, 1.34 is the O_2_ binding capacity of Hb and 0.003 is the solubility of O_2_ dissolved in blood.

Wall shear stress (ICA‐τ, dynes/cm^2^) was calculated as:

$$4\eta \;(P)\left(\frac{\mathrm{ICA}-V_{P\;(\mathrm{cm}/s)\;}}{\mathrm{ICA}-\mathrm{Diameter}\;(\mathrm{cm})}\right)$$

where η, whole blood viscosity at a shear rate of 225/s measured in vitro at 37°C; P, pois.

Cerebral substrate delivery [oxygen and glucose ($D_{O_{2}}$ and *D*_Glu_)] were estimated as:

$$\mathrm{ICA}-D_{O_{2}}\left(\mathrm{mL}/\min\right)=\left(\frac{{\dot{Q}}_{\mathrm{ICA}}\;(\mathrm{mL}/\min)\;}{100}\right)\times C_{aO_{2}}\left(\mathrm{mL}/\mathrm{dL}\right)$$

$$\mathrm{ICA}-D_{\mathrm{Glu}}\left(\mathrm{mmol}/\min\right)=\left(\frac{{\dot{Q}}_{\mathrm{ICA}}\;(\mathrm{mL}/\min\;}{1000}\right)\times \mathrm{Glucose}\;\left(\mathrm{mmol}/L\right)$$

#### 2.4.7 Statistical analyses

Statistical analyses were undertaken using SPSS for Windows (Version 29; IBM Corp., Armonk, NY, USA). Following confirmation of distribution normality using the Shapiro–Wilk *W*‐tests, differences in the cardiopulmonary response to CPET between the patient and participant [mean of nine measurements during exercise (seven data points) and recovery (two data points)] were determined using an independent samples Student's *t*‐test. Significance for all one‐tailed tests was established at *P* < 0.05. Data are expressed as means ± SD.

## 3 RESULTS

### 3.1 Cardiopulmonary responses to CPET (case and control)

Figure 2 highlights the patient's hypertensive response to CPET compared to the normotensive response in the healthy participant (mean MAP of 193 ± 31 vs. 139 ± 26 mmHg, *P*  = <0.001). The corresponding mean RPP was also markedly higher in the patient (231 ± 104 vs. 148 ± 88 mmHg × 10^2^ AU *P* = 0.044).

### 3.2 Recovery perfusion (Case)

Duplex scanning confirmed significant, albeit incomplete recovery of (left) ICA luminal calibre and consequent perfusion (Figure 5). Left $\dot{Q}$_CCA_, $\dot{Q}$_ICA_ and $\dot{Q}$_VA_ were 593, 447 and 96 mL/min, respectively and were generally lower compared to the right‐sided extracranial circulation (617, 591 and 129 mL/min, respectively, Figure 5).

### 3.3 Simulation sub‐study (Control)

#### 3.3.1 Cardiopulmonary function

As anticipated, pyramid breathing during simulated swimming (Figure 6) was more physiologically demanding compared to standard breathing as evidenced by more marked elevations in HR, $\dot{Q}$, MAP and corresponding TPR (Table 1). Intermittent apnoea also induced relative hypercapnia (elevated $P_{\mathrm{ETC}O_{2}}$) in the absence of arterial hypoxaemia (invariant $S_{pO_{2}}$, Table 1).

**TABLE 1:** Physiological responses to dry‐land static swimming.

	Rest	Swimming exercise	
State	Standard breathing	Standard breathing	Pyramid breathing
Cardiopulmonary			
HR (b/min)	51	91	107
SV (mL/min)	85	96	91
$\;\dot{Q}\;$(L/min)	4.3	8.7	9.7
MAP (mmHg)	83	96	105
TPR (mmHg/L/min)	19.3	11.0	10.8
$P_{\mathrm{ETC}O_{2}}$ (mmHg)	38	37	44
$S_{pO_{2}}$ (%)	97	97	97
Cerebrovascular			
ICA‐Velocity (cm/s)	36.1	38.1	40.1
ICA‐Diameter (cm)	0.462	0.468	0.473
$\;\dot{Q}$_ICA_ (mL/min)	181	197	211
ICA‐CVCi (mL/min/mmHg)	2.18	2.05	2.01
η (cP)	4.05	4.59	4.65
ICA‐τ (dynes/cm^2^)	12.7	14.9	15.8
ICA‐$D_{O_{2}}$ (mL/min)*	37.7	40.9	43.9
ICA‐D_Glu_ (mmol/min)**	0.87	0.94	1.01

Values are means ± SD based on a single healthy participant who remained prone across all three conditions. *Calculated based on a whole blood haemoglobin concentration of 15.8 g/dL. **Plasma glucose concentration of 4.8 mmol/L. cP, centipoise; CVCi, cerebrovascular conductance index; *D*_Glu_, glucose delivery; $D_{O_{2}}$, oxygen delivery; η, whole blood viscosity; HR, heart rate; MAP, mean arterial pressure; $P_{\mathrm{ETC}O_{2}}$, end‐tidal partial pressure of carbon dioxide; $\dot{Q}$, cardiac output; $\dot{Q}$_ICA_ internal carotid artery blood flow; $S_{pO_{2}}$, peripheral oxygen saturation; SV, stroke volume; τ, wall shear stress; TPR, total peripheral resistance.

#### 3.3.2 Cerebrovascular function

Table 1 highlights the integrated cardiopulmonary–cerebrovascular responses to the different exercise paradigms. As anticipated, the physiological stress of swimming exercise that incorporated pyramid breathing was greater resulting in greater increases in HR, $\dot{Q},$ MAP and $P_{\mathrm{ETC}O_{2}}$ compared to standard breathing (Table 1, Figure 6). The (exercise‐induced) elevation in $\dot{Q}$_ICA_ was also more pronounced due to increases in both ICA‐velocity and ICA‐diameter (Table 1). In combination with the general exercise‐induced elevation in η, pyramid breathing was accompanied by corresponding greater elevations in ICA‐τ_P_, ICA‐$D_{O_{2}}$ and ICA‐*D*_Glu_ compared to standard breathing (Table 1).

## 4 DISCUSSION

### 4.1 Clinical presentation

The presentation of ICA dissection documented in the present case report involved a combination of vague and more common symptoms including a popping sensation in the left ear, visual blurring, tinnitus and a unilateral, diffuse headache. The onset of these symptoms coincided acutely with recent completion of a strenuous swimming session that specifically incorporated pyramid breathing, with a left‐sided Horner's syndrome developing in the subsequent 48 h.

Horner's syndrome occurs when there is an interruption in the sympathetic chain somewhere along its course from the central brainstem to a peripheral postganglionic region. It is estimated to occur in approximately 25% of carotid artery dissection cases and is classified according to whether the causative lesion is central, pre‐ or post‐ganglionic. Horner's syndrome as a manifestation of carotid artery dissection is post‐ganglionic – affecting the neurones of the oculosympathetic pathway that ascend within the ICA adventitia traversing the cavernous sinus and join the ophthalmic division of the trigeminal nerve. These nerve fibres supply the pupillary sphincter and the levator palpebrae superioris (Reede et al., 2008). The dissection itself results in disruption of the arterial intima triggering oxidative–inflammatory–nitrosative stress and platelet activation that collectively converge to activate coagulation (Fall et al., 2015). This process can also result in vessel occlusion or distal embolisation. Hence, early antithrombotic therapy is recommended to reduce the risk of embolisation occurring (Morel et al., 2012). A more sinister consequence is pseudoaneurysm formation (exhibited in the patient's right ICA), which carries the risk of an expanding neck mass or free rupture. However, in the current case, this was considered safe to be left alone.

### 4.2 Pathophysiology and mechanisms

Isolating the precise mechanisms and corresponding risk factors that caused the ICA dissection in the present case report is challenging. Clinical investigations excluded the presence of any pre‐existing, albeit rare (<1%), connective tissue disorders including Marfan, Ehlers–Danlos and Loeys–Dietz syndromes (Debette et al., 2014) or connective tissue weakness defined by joint hypermobility, thin and translucent skin, or easy bruising, which collectively associate with carotid artery dissection (Giossi et al., 2014). Echocardiography confirmed a normal (i.e. healthy) aortic root. Renal arteries were also healthy (MRA) with no evidence of fibromuscular dysplasia.

The cause of the patient's hypertension is unknown and may be essential, although it responded well to the combination of medication and a low carbohydrate diet. It was also of interest to note the pseudoaneurysm in the distal right ICA, which was considered a sequela of a previous dissection, indicating that the presenting symptoms were due to a separate (i.e., second) dissection. This was likely asymptomatic since there were no prior localising or ischaemic symptoms the patient was aware of at the time, including normal coronary arteries on CT. While a post‐carotid artery dissection aneurysm can develop, the consensus is that these follow a benign course and are not associated with an increased risk of subsequent stroke (Debette et al., 2021; Lee et al., 2006).

A causal link between carotid artery dissection and physical activity remains controversial (Engelter, Tranenka, Grond‐Ginsbach et al., 2021). However, given that the onset of the patient's symptoms coincided with a recent bout of dynamic intermittent apnoeic (pyramid breathing) swimming, we tentatively suggest that this specific mode of exercise may have been a precipitating factor. The mechanical stress of front crawl swimming involving abrupt rotation and occasional rapid flexion–extension of the neck combined with (exercise‐induced) elevations in MAP has been identified as a potential risk factor for carotid artery dissection (Schlemm et al., 2017). While the pyramid breathing swimming that the patient engaged with in this case report would have incurred fewer rotations compared to standard breathing, it is feasible that bilateral, as opposed to unilateral rotations constrained to his favoured right‐side (contralateral to the ICA dissection), would have invoked greater mechanical torsion of an asymmetrically adapted ICA.

Importantly, inclusion of the simulation sub‐study involving an age‐, sex‐ and activity‐ matched healthy control participant provided proof‐of‐principle evidence to suggest that pyramid breathing conferred greater cerebrovascular risk compared to standard breathing. We observed a more marked (simulated) swimming‐induced elevation in $\dot{Q}$_ICA_. This was likely attributable to an apnoea‐induced greater elevation in sympathetic outflow (Steinback et al., 2010), MAP (Breskovic et al., 2011) and relative hypercapnia (Bailey et al., 2024) to which the cerebrovasculature has evolved exquisite sensitivity (Bailey, 2019a, 2019b; Bailey et al., 2017) – with little to no contribution from hypoxaemia‐induced pial artery dilatation since the intermittent apnoeas were clearly too short to effect systemic arterial desaturation.

In combination with an exercise‐induced systemic oxidative–inflammatory–nitrosative stress‐mediated increase in η (Fall et al., 2018, 2015), this evoked a more pronounced elevation in τ_P_ that may have further compromised structural integrity of the ICA wall (Bailey et al., 2023). Further elevations in $\dot{Q}$_ICA_ and τ_P_ would have been expected during water immersion (which we were not logistically equipped to replicate) subsequent to: (1) intermittent Valsalva manoeuvres caused by interrupted breathing eliciting transient elevations in intrathoracic pressure‐induced MAP (phase I) (Tiecks et al., 1995) and (2) hydrostatic pressure‐induced centralised redistribution of blood volume (Pendergast et al., 2015) that in accordance with the Frank–Starling mechanism would have been expected to cause further elevations in cardiac preload, SV and MAP, and (more) pronounced hypercapnia caused by systemic CO_2_ retention subsequent to improved ventilation–perfusion matching (Serrador et al., 2006; Shoemaker et al., 2019). Collectively, these findings highlight the inherent risks and misguided belief that this constitutes a safe and effective mode of intermittent ‘hypoxic’ swim training, questioning its practice especially in susceptible individuals such as those with hypertension or prior vascular pathologies and abnormalities.

### 4.3 Management and recovery

Due in part to its rarity, there is no specific classification system for carotid artery dissection. However, a system derived from the classification of blunt carotid artery trauma may be applicable. This system classifies blunt carotid injury into four types specifically related to the mechanism of injury. This ranges from a direct vascular injury to complications arising from hyperextension (Crissey & Bernstein, 1974). The Denver classification subsequently refined this scheme and examined the consequences of the initiating event on the artery itself. A grade 1 injury is classified as an intimal irregularity or dissection with <25% luminal narrowing whilst grade 5 is a complete vessel transection and free extravasation of blood (Biffl et al., 2001).

The appropriate management for these lesions differs considerably. Grade 5 is considered to have a high mortality risk and even if initially survived, a cerebrovascular event is a likely outcome. Grade 1 lesions can be successfully treated with up to 6 months of oral anticoagulant (OAC) or antiplatelet (APT) therapy. For the higher‐grade lesions, OAC or APT is probably insufficient and either surgery or an endovascular technique (see below) should also be considered. If embolisation has occurred into the distal cerebral vasculature, mechanical thrombectomy has been demonstrated safe and effective with improved outcomes documented compared to medications alone (Dmytriw et al., 2020; Markus et al., 2015; Yaghi, Engelter et al., 2024).

The issue of optimum medical therapy was investigated in the CADISS Trial (Markus et al., 2015). This study assessed the use of APT against OAC in preventing stroke following both carotid and vertebral artery dissection. The findings demonstrated no difference between the groups with the overall risk of stroke classified as low. To the best of the current authors’ knowledge, there has only been one other randomised controlled trial (TREAT) that compared APT with OAC in patients with symptomatic carotid artery dissection. This trial demonstrated that all strokes occurred in the APT group, with all events occurring within 7 days of initial randomisation (Engelter, Traenka, Gensicke et al., 2021).

The remaining evidence is based on a combination of prospective and retrospective studies that explored the optimum management of carotid artery dissection. The STOP‐CAD study investigated 3636 patients and demonstrated that those taking OAC following occlusive dissection exhibited a lower risk of stroke (Yaghi, Shu et al., 2024). The authors concluded that OAC should be instigated within the first month following dissection followed by APT, due to the higher risk of haemorrhage with OAC. Furthermore, the risk of recurrent ischaemia is highest during the first 2–4 weeks following the initiating dissection (Pini et al., 2024). The risk of stroke by 6 months was significantly decreased, and the conclusion was that antithrombotic therapy should be commenced as soon as possible following the carotid artery dissection and continued for 6 months duration.

Only four prospective studies have been documented in the literature (Albuquerque et al., 2002; Juszkat et al., 2015; Pezzini et al., 2022; Yaghi, Shu et al., 2024) with two dealing solely with post‐traumatic carotid artery dissection (Albuquerque et al., 2002; Juszkat et al., 2015). As far as invasive endovascular techniques such as carotid stenting are concerned, current guidelines recommend stenting in those with recurrent neurological symptoms even though they may be prescribed optimum medical therapy (Cohen et al., 2012). Recurrent neurological symptoms in this situation are attributable to cerebral hypoperfusion, which may occur with or without a thromboembolic event. The use of an intraluminal stent has the proviso that there should be evidence of salvageable cerebral tissue upon reperfusion. Specialist neuroimaging may be able to predict those with carotid artery dissection at risk of recurrent events whilst on optimum medical therapy. There are several case reports and retrospective studies concerning the use of stents for carotid artery dissection.

Carotid artery dissections do not usually cause distal cerebral hypoperfusion when a stenosis or occlusion occurs. If a carotid artery dissection has caused cerebral ischaemia (due to either vessel occlusion or embolisation), the clinical picture is dependent on the circle of Willis and collateralisation. Many patients will recover with little to no intervention, and this should be a consideration when a carotid stent might be an option, since it will improve vessel patency but not necessarily cerebral function (Touze et al., 2001). This is reflected by the lack of evidence for routine stent placement even after an embolic event (Yaghi, Engelter et al., 2024). With the widespread extra‐ and intracranial neuroimaging (from aortic arch to vertex) routinely available, it is likely that increasing numbers of carotid artery dissections will be detected. Perfusion scans can detect early cerebral ischaemia, and there may be more consideration for early intervention. The use of stents and thrombectomy has an uncertain place in the management of carotid artery dissection, whereas APT is now generally first line with OAC reserved for certain cases (free‐floating thrombus and significant embolic risks) as highlighted.

In this case–control report, we observed significant re‐expansion of the true lumen of the ICA at the skull base, although duplex imaging indicated that perfusion was not fully restored compared to the (control) right‐sided circulation implying some degree of arterial remodelling. There is evidence that this can occur in ∼25% of cases within a median time of approximately 4 months (Coldwell et al., 2000). The altered diet and graduated, structured return to exercise training and competitive racing proved to be important parts of the patient's recovery process. Swimming exercise, while avoiding pyramid breathing and exaggerated elevations in ICA shear, similar to the avoidance of weight training‐induced Valsalva manoeuvres for spontaneous coronary artery dissections (Hayes et al., 2018), continues to be well tolerated by the patient, who remains free of any related neurological sequelae.

### 4.4 Conclusion

The present case–control study provided a unique opportunity to document the preceding events, clinical presentation, pathophysiology and treatment of ICA dissection, a rare cause of cerebral ischaemia. The patient's symptoms coincided with a recent bout of dynamic intermittent apnoeic (pyramid breathing) swimming that follow‐up work conducted during a dry‐land static swimming simulation study tentatively suggests may have been a precipitating factor, potentially related to exaggerated ICA shear stress. Our findings emphasise the importance of considering a patient's history of recent trauma or connective tissue disorder as part of the differential diagnosis in stroke management, and that early repeat imaging is performed once carotid artery dissection is diagnosed, particularly if subtle changes in symptoms occur or if there is a known unstable lesion in the carotid artery. Furthermore, they highlight the inherent risks and misguided belief that this constitutes a safe and effective mode of intermittent ‘hypoxic’ swim training, questioning its practice especially in susceptible individuals such as those with hypertension or prior vascular pathologies and abnormalities. Finally, registries should be considered, to help develop flow charts of appropriate therapeutic options (invasive treatment or optimum medical therapy) and improve clinical outcomes.

## AUTHOR CONTRIBUTIONS

Damian M. Bailey conceived the idea and wrote the first draft of the manuscript with input from Ian M. Williams, Richard G. Davies, Brenig L. Gwilym and Richard D. White. Damian M. Bailey, Richard G. Davies, Brenig L. Gwilym, Benjamin S. Stacey, Danny Walmsley, Michael H. Lewis, Josip Butkovic, Zvonomir Vrselja, Shigehiko Ogoh, James A. Pawelczyk, Mohamad Bashir, Richard D. White and Ian M. Williams edited and revised the manuscript. Damian M. Bailey, Richard G. Davies, Brenig L. Gwilym, Benjamin S. Stacey, Danny Walmsley, Michael H. Lewis, Josip Butkovic, Zvonomir Vrselja, Shigehiko Ogoh, James A. Pawelczyk, Mohamad Bashir, Richard D. White and Ian M. Williams approved the final version submitted for publication and agree to be accountable for all aspects of the work in ensuring that questions related to the accuracy or integrity of any part of the work are appropriately investigated and resolved. All persons designated as authors qualify for authorship, and all those who qualify for authorship are listed.

## CONFLICT OF INTEREST

D.M.B. is Editor‐in‐Chief of *Experimental Physiology*, Chair of the Life Sciences Working Group, member of the Human Spaceflight and Exploration Science Advisory Committee to the European Space Agency, member of the Space Exploration Advisory Committee to the UK and Swedish National Space Agencies and member of the National Cardiovascular Network for Wales and South‐East Wales Vascular Network. D.M.B. is also affiliated to Bexorg, Inc. (USA) focused on the technological development of novel biomarkers of cerebral bioenergetic function and structural damage in humans. I.M.W. is a member of the South‐East Wales Vascular Network. M.B. is a Consultant for Terumo & JOTEC/CryoLife.

## Data Availability

The data that support the findings of this study are available from the corresponding author upon reasonable request.
